# Editorial: Diagnosing and treating frailty and sarcopenia in middle-aged and older adults

**DOI:** 10.3389/fspor.2025.1736677

**Published:** 2025-11-24

**Authors:** Diogo L. Marques, Henrique P. Neiva, Daniel A. Marinho, Mikel Izquierdo, Mário C. Marques

**Affiliations:** 1 Department of Sport Sciences, University of Beira Interior, Covilhã, Portugal; 2 Research Centre in Sport Sciences, Health Sciences, and Human Development (CIDESD), Covilhã, Portugal; 3 Navarrabiomed, Hospital Universitario de Navarra (HUN) – Universidad Pública de Navarra (UPNA), IdiSNA, Pamplona, Spain; 4 CIBER of Frailty and Healthy Aging (CIBERFES), Instituto de Salud Carlos III, Madrid, Spain

**Keywords:** screening, sarcopenia, muscle strength, physical performance, exercise, aging

In light of the global population's increasing age, it is essential to thoroughly explore the challenges this presents, especially within healthcare systems. As individuals age, various physiological and structural changes can affect their ability to generate force, often leading to a decline in functional capacity. Consequently, geriatric syndromes such as frailty and sarcopenia are becoming increasingly prevalent (1, 2). These conditions are interconnected and linked to numerous adverse outcomes, including an increased risk of falls, hospitalization, and even death. These outcomes significantly affect the quality of life for older adults (3, 4).

In this Research Topic, we have collected 10 research articles that examine the use of valid testing protocols for accurate diagnosis of frailty and sarcopenia and the application of effective interventions to treat or reverse these conditions. These geriatric research articles offer valuable insights into systems and cutoff values for various populations and age groups, along with the development of more effective, individualized exercise programs. Consequently, these advancements allow for the identification of optimal strategies to preserve functional capacity in older populations.

In this context, the study by Setiati et al. provided valuable normative data, establishing crucial cutoff values for calf circumference, muscle strength, and physical performance indicators for diagnosing sarcopenia in Indonesian and Asian populations. Their multicenter, cross-sectional study involved 905 healthy Indonesian adults aged 20–39 years in urban regions. The authors used a 2-standard-deviation cut-off from the mean of a healthy young adult population to define normality. The following cut-off points were proposed: low calf circumference <29.92 cm and <26.70 cm; low handgrip strength <21.15 kg and <14.34 kg for men and women, respectively. Moreover, low physical performance was indicated by a walking pace of <0.51 m·s^−1^.

Wei et al. explored the relationship between chronic diseases, lifestyle factors, and the risk of sarcopenia among older Chinese individuals. Their research used data from the China Health and Retirement Longitudinal Study (CHARLS) on participants aged 60 years and older (*n* = 7,841). The researchers found a non-linear relationship between physical activity and the risk of sarcopenia, and that the primary risk factors for sarcopenia include advancing age, chronic health conditions, lifestyle habits, and levels of social participation.

In a similar vein, but focusing only on a rural Chinese population aged 60–94 years (*n* = 291), Guo and Shi sought to identify risk factors for physical frailty by developing a predictive model, discovering that family income, physical activity, depressive symptoms, and fear of falling are significant risk factors for physical frailty in rural older adults, underscoring the importance of identifying high-risk environments to prevent frailty.

Given that machine learning (ML) is a powerful tool for predictive disease modeling, three studies were conducted to explore its applications. First, Du et al. also used CHARLS data to identify risk factors for sarcopenia (*n* = 2,717). ML allowed for the identification of time-dependent risk factors. The XGB algorithm was used, with an area under the receiver operating characteristic curve (ROC-AUC) of 0.70. These cohort study findings showed that living conditions, health status, and behavioral habits significantly influenced the model's predictive performance.

In another ML exploration using the CHARLS dataset, Peil Yu et al. examined community-dwelling older adults with cardiovascular disease (CVD). A sarcopenia prevalence of 18.61% was identified. The XGB algorithm was used, with an ROC-AUC of 0.87. The authors' findings indicated that, in older adults with CVD, lower BMI was a predictor of sarcopenia; conversely, regular weekly contact with children was found to act as an important protective factor.

Yu et al. investigated the relationship between grip strength and frailty using the CHARLS dataset (*n* = 10,834). The LightGBM model was used, with an ROC-AUC of 0.77. The researchers identified a significant negative correlation between grip strength and frailty risk, with variation based on gender (especially above 29.00 kg for men and 19.00 kg for women).

Given that insulin resistance and sarcopenia are prevalent among older adults, Li et al. conducted a study to investigate the correlations between the triglyceride-glucose (TyG) index, the TyG-body mass index (TyG-BMI), and sarcopenia in Chinese non-diabetic middle-aged and older women. Their cross-sectional study included a sample of 460 postmenopausal women and revealed a significant association between a higher TyG index and TyG-BMI index and a reduced risk of sarcopenia. This finding underscores the value of considering the TyG-BMI index as an effective biomarker for predicting sarcopenia risk in this population.

Supporting the idea that resistance training programs are a vital intervention to combat frailty in older adults living in daycare centers, Abreu et al. investigated, in a 12-week interventional study, the effects of a low-volume, remotely supervised resistance training (RT) program in four Portuguese daycare centers (*n* = 44). The prevalence of all frailty criteria decreased during the intervention period (from 48.4% to 25.8%), with the most significant reductions observed in low physical activity and exhaustion. This approach demonstrated that a low-volume, remotely supervised RT program can provide substantial benefits in the management of frailty.

In line with the importance of analyzing exercise approaches to counteract age-related muscle weakness, Desachy et al. conducted a comprehensive analysis comparing cortical activity during concentric and eccentric quadriceps contractions in both young and older healthy participants (*n* = 38). The authors found that eccentric movements elicited greater cortical activity than concentric movements in both age groups. This finding is particularly valuable for effectively addressing age-related issues, as it targets both muscle atrophy and brain changes.

Finally, within a patient-centered framework, Uchmanowicz et al. emphasized the significance of a multidisciplinary, multi-specialty, and holistic approach to frailty interventions. This framework encourages collaboration among professionals from different fields to enhance patients’ overall quality of life. The authors point out that dietary, exercise, and cognitive interventions must be personalized for everyone, while simultaneously promoting opportunities for social engagement.

The collection of articles in this Research Topic aims to contribute to the advancement of research in geriatrics by encouraging the development of practical interventions and strategies for the early diagnosis and treatment of frailty and sarcopenia in middle-aged and older adults.

## References

[1] Izquierdo M De Souto Barreto P Arai H Bischoff-Ferrari HA Cadore EL Cesari M Global consensus on optimal exercise recommendations for enhancing healthy longevity in older adults (ICFSR). J Nutr Health Aging. (2025) 29:100401. 10.1016/j.jnha.2024.100401 39743381 PMC11812118

[2] Ye L Liang R Liu X Li J Yue J Zhang X. Frailty and sarcopenia: a bibliometric analysis of their association and potential targets for intervention. Ageing Res Rev. (2023) 92:102111. 10.1016/j.arr.2023.102111 38031836

[3] Fragala MS Cadore EL Dorgo S Izquierdo M Kraemer WJ Peterson MD Resistance training for older adults: position statement from the national strength and conditioning association. J Strength Cond Res. (2019) 33:2019–52. 10.1519/jsc.0000000000003230 31343601

[4] Dent E Morley JE Cruz-Jentoft AJ Woodhouse L Rodríguez-Mañas L Fried LP Physical frailty: ICFSR international clinical practice guidelines for identification and management. J Nutr Health Aging. (2019) 23:771–87. 10.1007/s12603-019-1273-z 31641726 PMC6800406

